# New positive patch test reactions on day 7—The additional value of the day 7 patch test reading

**DOI:** 10.1111/cod.13322

**Published:** 2019-06-11

**Authors:** Cynthia C. A. van Amerongen, Robert Ofenloch, Daan Dittmar, Marie L. A. Schuttelaar

**Affiliations:** ^1^ Department of Dermatology University of Groningen, University Medical Centre Groningen Groningen The Netherlands; ^2^ Department of Social Medicine, Occupational and Environmental Dermatology University of Heidelberg Heidelberg Germany

**Keywords:** allergic contact dermatitis, D7 reading, epidemiology, new positive D7 reactions, patch testing

## Abstract

**Background:**

Not performing a day (D) 7 patch test reading might result in positive patch test reactions being missed.

**Objectives:**

To investigate the added value of the D7 patch test reading for individual allergens, and to identify patient characteristics and allergen groups associated with new positive D7 reactions.

**Methods:**

Data from patients patch tested between 2008 and 2018 with the extended European baseline series were analysed. Patch test readings were performed on D3 and D7. Positive reactions were categorized into positive on D3 or new positive on D7.

**Results:**

A total of 3292 patients were consecutively patch tested with at least 43 allergens of the TRUE Test panels 1 and 2 supplemented with investigator‐loaded allergens. In total, 447 (13.6%) patients showed new positive D7 reactions. In univariable regression analysis, age between 18 and 30 years showed a negative association with new positive D7 reactions. Significantly more D7 positive reactions were seen for topicals (odds ratio [OR] 2.60, 95% confidence interval [CI]: 1.92‐3.51) and corticosteroids (OR 1.87, 95%CI: 1.09‐3.21). No associations were found between sex, atopic dermatitis and occupational dermatitis and a new positive D7 reaction.

**Conclusion:**

A D7 reading to identify new positive patch test reactions is of added value, especially for topicals and corticosteroids.

## 1 INTRODUCTION

According to the European Society of Contact Dermatitis (ESCD) guideline for diagnostic patch testing, it is recommended to perform at least two readings, on day (D) 2, D3, or D4, and around D7.^1^ Most centres perform patch test readings on D2 to D4. A late patch test reading on D7 in addition to the D3 or D4 reading can identify new positive patch test reactions, ranging from 3% up to 34% in previous studies, which were found to be negative, doubtful or irritant on preceding readings.^2^, ^3^, ^4^, ^5^, ^6^, ^7^, ^8^, ^9^ Multiple studies have reported the importance of an additional late patch test reading, especially for suspected contact allergies to metals, topicals, and corticosteroids. However, the allergens that have shown new positive reactions at an additional late patch test reading vary in the literature.^7^, ^10^, ^11^ It is unknown whether there are associated factors, for example, patient characteristics, that may enhance the tendency to develop new positive reactions at a D7 reading. Only one study evaluated sex and age as possible associated factors, and found a significantly higher rate of new positive D7 reactions in women than in men and in patients aged >40 years.^7^ The objectives of the current study were to evaluate the added value of the D7 patch test reading for individual allergens and allergen groups, and to identify factors associated with new positive D7 reactions.

## 2 METHODS

### 2.1 Study design and patch testing

We performed a retrospective data analysis on patch test data collected between January 2008 and July 2018 for consecutively patch tested patients who were routinely tested with our extended European baseline series. TRUE Test panels 1 and 2 (SmartPractice Europe, Reinbek, Germany) supplemented with additional investigator‐loaded allergens (SmartPractice Europe, and Chemotechnique Diagnostics, Vellinge, Sweden) tested in Van der Bend square chambers (Van der Bend, Brielle, The Netherlands) were applied on the back for 48 hours under occlusion. Patch test readings were performed on D3 and D7. The readings were performed by experienced dermatologists according to the ESCD guideline.^1^ Weak (+), strong (++) and extreme (+++) reactions were classified as positive reactions. Reactions reported as irritant, doubtful (?+) or follicular were counted as negative reactions in addition to the negative results. In addition, patch test results reported as irritant, doubtful or follicular at the D3 reading that became positive on D7 were evaluated separately. Reactions were evaluated as irritant if margins were sharply demarcated and the surface of the test area showed a silk paper structure or a shiny skin. Reactions were considered to be doubtful if erythema and/or infiltration did not cover the whole test area.^12^

### 2.2 Data analysis

Positive reactions were categorized into positive on D3 or new positive on D7 (labelled as negative on D3). For patients who had been tested more than once, only the most recent patch test result was evaluated. To evaluate patient characteristics, three groups of patients were defined: patients with only D3 positive reactions and no new positive D7 reactions; patients with only new D7 positive reactions corresponding to D3 negative reactions; and patients with both D3 positive reactions and new D7 positive reactions. The patient characteristics that were analysed were age, sex, atopic dermatitis in the patient's lifetime, and occupational dermatitis. Age was categorized into the following groups: <18, 19 to 30, 31 to 45, 46 to 60, and ≥61 years. To analyse the influence of type of allergen, allergens were grouped on the basis of chemical structure, cross‐reactivity, and co‐sensitization, resulting in seven groups: metals, preservatives, fragrances, rubbers, dyes, topical medicaments ("topicals"), and corticosteroids (Table 1).^13^, ^14^ A positive reaction to at least one of the allergens in the group was counted as an overall positive group reaction.

**Table 1:** Overview of the composition of each allergen group used for univariable regression analysis

Allergen group	Composition
Metals	Nickel sulfate, potassium dichromate, cobalt chloride
Preservatives	Paraben mix, MCI/MI, quaternium‐15, formaldehyde, thiomersal, MI, MDBGN, 2‐bromo‐2‐nitropropane‐1,3‐diol, diazolidinyl urea, imidiazolidinyl urea, 1,2‐benzisothiazolin‐3‐one
Fragrances	FM I, *Myroxylon pereirae* resin (balsam of Peru), FM II, HICC
Rubber additives	Carba mix, black rubber mix, MBT, mercapto mix, thiuram mix
Dyes	*p*‐Phenylenediamine, textile dye mix, Disperse Blue 106, Disperse Blue 124, toluene‐2,5‐diamine
Topicals	Neomycin sulfate, lanolin alcohols, caine mix, quinoline mix, sesquiterpene lactone mix, parthenolide, Amerchol L 101
Corticosteroids	Budesonide, tixocortol pivalate, hydrocortisone‐17‐butyrate

Abbreviations: FM I, fragrance mix I; FM II, fragrance mix II; HICC, hydroxyisohexyl 3‐cyclohexene carboxaldehyde (Lyral); MBT, mercaptobenzothiazole; MCI, methylchloroisothiazolinone; MDBGN, methyldibromo glutaronitrile; MI, methylisothiazolinone.

### 2.3 Statistics

Descriptive data are presented in tables as numbers with percentages and 95% confidence intervals (CIs). Prevalences are presented as both crude and age‐standardized estimates with accompanying 95%CIs. The European standard population of 2013 was used as the reference for age and sex standardization.^15^ Univariable logistic regression was performed to analyse the association between patient characteristics and allergen groups on new positive D7 reactions, and these were expressed as odds ratio (ORs) with 95%CIs. Statistical analyses were performed with spss v.23 (IBM, Armonk, New York) and Excel 2013 (Microsoft, Redmond, Washington).

## 3 RESULTS

### 3.1 New D7 positive patch test reactions

A total of 3292 patients (67.0% female, 33.0% male, mean age 42.7 ± 16.9 years) were consecutively patch tested with at least 43 allergens of the TRUE Test panels 1 and 2 supplemented with investigator‐loaded allergens. The sociodemographic characteristics of the total patch tested group and the subgroup of patients with at least one positive reaction are shown in Table 2. A total of 1653 patients (50.2%) had at least one positive reaction on D3 and/or D7. Of the total 3292 patch tested patients, 189 (5.7%) showed only new positive D7 reactions (ie, read as negative, doubtful or irritant on D3) and another 258 (7.8%) patients showed positive D3 reactions with additional new positive D7 reactions. Thus, in total, 447 (13.5%) of the patch tested patients had new positive D7 patch test reactions. Both crude and age‐standardized and sex‐standardized prevalences of the total number of positive reactions on D3 and D7 are shown together with 95%CIs in Table 3. The allergens with the highest proportions of new positive D7 reactions (new positive D7 reactions/total positive reactions) in order of frequency were neomycin sulfate (81.5%, 22/27 patients), 2‐bromo‐2‐nitropropane‐1,3‐diol (50%, 11/22 patients), budesonide (42.3%, 11/26 patients) and diazolidinyl urea (41.4%, 12/29 patients). For groups of allergens, the proportions of new positive D7 reactions were, in order of decreasing frequency, most frequent for topicals (33.3%, 72/216 patients), corticosteroids (28.4%, 19/67 patients), dyes (20.2%, 53/263 patients), fragrances (16.6%, 95/572), metals (16.3%, 147/904), preservatives (15.1%, 113/749), and rubber additives (7.6%, 23/304).

**Table 2:** Sociodemographic characteristics of the patch tested population

	Total tested (N = 3292)			Reacted positively on D3 and/or D7 (N = 1653)		
	n	%	95% CI	n	%	95% CI
Sex						
Male	1087	33.0	31.4‐34.6	453	27.4	25.3‐29.6
Female	2205	67.0	65.4‐68.6	1200	72.6	70.4‐74.7
Age (y)						
<18	112	3.4	2.8‐4.0	42	2.5	1.8‐3.3
18‐30	918	27.9	26.4‐29.4	384	23.2	21.2‐25.3
31‐45	787	23.9	22.4‐25.4	425	25.7	23.6‐27.8
46‐60	912	27.7	26.2‐29.2	525	31.8	29.5‐34.0
≥61	563	17.1	15.8‐18.4	277	16.8	15.0‐18.6
Atopic dermatitis (lifetime prevalence)						
Yes	1409	42.8	41.1‐44.5	701	42.4	40.0‐44.8
No	1883	57.2	55.5‐58.9	952	57.6	55.2‐60.0
Occupational dermatitis						
Yes	715	21.7	20.3‐23.1	388	23.5	21.4‐25.5
No	2577	78.3	76.9‐79.7	1265	76.5	74.5‐78.6

CI, confidence interval.

**Table 3:** Patch test results obtained with the baseline series with crude and age‐standardized and sex‐standardized prevalences of positive reactions on day (D) 3 and/or D7 accompanied by 95% confidence intervals (CIs)

								New positive D7 reactions								
		Total patch tested patients (n = 3292)	Total crude positives on D3 and/or D7 (n = 1653)		Total age‐standardized and sex‐standardized positives on D3 and/or D7			Total		Reaction type at D3 reading (n)			Reaction strength on D7 (n)			Proportion of total positive reactions
Haptens	Concentration (μg/cm^2^)	N	n	%	n	%	95% CI	n	%	−	?	IR	+	++	+++	%
Nickel sulfate	200	3172	607	19.1	424	14.5	12.2‐14.6	104	3.3	64	22	18^a^	96	8	0	17.1
Lanolin alcohols	1000	3218	44	1.4	41	1.4	0.9‐1.7	10	0.3	4	6	0	9	1	0	22.7
Neomycin sulfate	230	3209	27	0.8	25	0.9	0.5‐1.1	22	0.7	21	1	0	20	2	0	81.5
Potassium dichromate	23	3206	122	3.8	112	3.8	2.9‐4.1	18	0.6	11	7	0	16	2	0	14.8
Caine mix	630	3217	34	1.1	27	0.9	0.5‐1.2	9	0.3	9	0	0	7	2	0	26.5
Fragrance mix I	430	3216	138	4.3	115	3.9	2.9‐4.2	21	0.7	16	4	1	21	0	0	15.2
Colophonium	850	3204	110	3.4	95	3.2	2.4‐3.6	17	0.5	15	2	0	15	2	0	15.5
Epoxy resin	50	3216	91	2.8	92	3.1	2.3‐3.4	28	0.9	20	7	1	24	3	1	30.8
Quinoline mix	190	3218	7	0.2	9	0.3	0.1‐0.5	2	0.1	2	0	0	1	1	0	28.6
*Myroxylon pereirae* (balsam of Peru)	800	3217	89	2.8	59	2.0	1.4‐2.3	19	0.6	15	3	1	19	0	0	21.3
Ethylenediamine dihydrochloride	50	3218	29	0.9	34	1.2	0.7‐1.4	3	0.1	1	1	1^a^	3	0	0	10.3
Cobalt chloride	20	3211	175	5.5	160	5.4	4.2‐5.7	25	0.8	17	6	2^a^	23	2	0	14.3
PTBP‐FR	40	3207	92	2.9	78	2.7	1.9‐3.0	17	0.5	11	6	0	16	1	0	18.5
Parabens mix	1000	3218	9	0.3	9	0.3	0.1‐0.5	2	0.1	1	1	0	2	0	0	22.2
Carba mix	250	3217	117	3.6	125	4.2	3.2‐4.6	4	0.1	4	0	0	4	0	0	3.4
Black rubber mix	75	3218	34	1.1	28	0.9	0.5‐1.2	7	0.2	6	1	0	7	0	0	20.6
MCI/MI	4	3219	331	10.3	312	10.6	8.7‐10.7	23	0.7	18	5	0	20	3	0	6.9
Quaternium‐15	100	3218	61	1.9	45	1.5	1.0–1.8	15	0.5	12	3	0	14	1	0	24.6
Mercaptobenzothiazole	75	3217	34	1.1	34	1.2	0.7‐1.4	1	0.0	1	0	0	1	0	0	2.9
*p*‐Phenylenediamine	90	3210	105	3.3	83	2.8	2.0–3.1	13	0.4	7	6	0	11	2	0	12.4
Formaldehyde	180	3218	51	1.6	38	1.3	0.8‐1.6	13	0.4	8	5	0	11	2	0	25.5
Mercapto mix	75	3217	43	1.3	46	1.6	1.0‐1.8	2	0.1	1	1	0	2	0	0	4.7
Thiomersal	8	3209	40	1.2	30	1.0	0.6‐1.3	9	0.3	7	2	0	9	0	0	22.5
Thiuram mix	25	3207	76	2.4	73	2.5	1.8‐2.8	9	0.3	6	3	0	9	0	0	11.8
MI	0.2% aq.	466	32	6.9	31	7.2	4.4‐8.9	3	0.6	2	1	0	2	1	0	9.4
Fragrance mix II	14% pet.	3275	246	7.5	181	6.0	4.7‐6.3	33	1.0	14	18	1	33	0	0	13.4
HICC	5% pet.	3242	98	3.0	72	2.4	1.7‐2.7	22	0.7	17	5	0	22	0	0	22.4
Sesquiterpene lactone mix	0.1% pet.	3277	43	1.3	40	1.3	0.8‐1.6	13	0.4	10	3	0	11	2	0	30.2
Parthenolide	0.1% pet.	3233	42	1.3	37	1.2	0.8‐1.5	14	0.4	12	2	0	9	5	0	33.3
MDBGN	0.5% pet.	3247	127	3.9	134	4.5	3.4‐4.8	17	0.5	7	10	0	17	0	0	13.4
Budesonide	0.1% pet.	3276	26	0.8	22	0.7	0.4‐1.0	11	0.3	7	4	0	9	2	0	42.3
Tixocortol‐21‐pivalate	0.1% pet.	3277	27	0.8	29	1.0	0.6‐1.2	4	0.1	2	2	0	4	0	0	14.8
Hydrocortisone‐17‐butyrate	1% eth.	3250	14	0.4	12	0.4	0.2‐0.6	4	0.1	2	2	0	4	0	0	28.6
Textile dye mix	6.6% pet.	991	52	5.2	47	5.0	3.4‐6.1	12	1.2	8	4	0	11	1	0	23.1
Disperse Blue 106	1% pet.	3250	31	1.0	27	0.9	0.5‐1.1	9	0.3	8	1	0	9	0	0	29.0
Disperse Blue 124	1% pet.	3274	27	0.8	23	0.8	0.4‐1.0	10	0.3	10	0	0	10	0	0	37.0
Toluene‐2,5‐diamine	1% pet.	2333	48	2.1	35	1.6	1.0‐2.0	9	0.4	7	2	0	8	1	0	18.8
Sodium disulfite	1% pet.	395	35	8.9	35	9.6	6.1‐11.7	6	1.5	2	4	0	6	0	0	17.1
2‐Bromo‐2‐nitropropane‐1,3‐diol	0.5% pet.	3250	22	0.7	22	0.7	0.4‐1.0	11	0.3	7	4	0	11	0	0	50.0
Diazolidinyl urea	2% pet.	3250	29	0.9	21	0.7	0.4‐0.9	12	0.4	9	3	0	11	1	0	41.4
Imidiazolidinyl urea	2% pet.	3250	26	0.8	20	0.7	0.3‐0.9	7	0.2	3	4	0	6	1	0	26.9
1,2‐Benzisothiazolin‐3‐one	0.05% pet.	3230	21	0.7	25	0.8	0.5‐1.1	2	0.1	0	2	0	2	0	0	9.5
Amerchol L 101	50% pet.	606	19	3.1	14	2.5	1.1‐3.5	2	0.3	1	1	0	2	0	0	10.5

Abbreviations: HICC, hydroxylisohexyl 3‐cyclohexene carboxaldehyde (Lyral); IR, irritant reaction; MCI, methylchloroisothiazolinone; MDBGN, methyldibromo glutaronitrile (dibromodicyanobutane); MI, methylisothiazolinone; PTBP‐FR, *p‐tert*‐butylphenol formaldehyde resin.

a Follicular reactions: nickel sulfate, n = 18; ethylenediamine dihydrochloride, n = 1; cobalt chloride, n = 2.

### 3.2 Regression analysis

A univariable logistic regression analysis was performed to investigate patient characteristics and allergen groups possibly associated with the occurrence of new positive D7 reactions. The results are shown in Table 4. For age, a significant negative association was found in the age group 18 to 30 years as compared with the age group >61 years (OR 0.58, 95% CI: 0.36‐0.93). No significant associations were found for sex, atopic dermatitis in the patient's lifetime and occupational dermatitis and a new positive D7 reaction. Significant associations were found for the allergen groups of topicals (OR 2.60, 95% CI 1.92‐3.51) and corticosteroids (OR 1.87, 95% CI 1.09‐3.21) and a new positive D7 reaction. Rubbers showed the lowest prevalence (7.6%) of new positive D7 reactions, and were significantly negatively associated with new positive D7 reactions (OR = 0.37, 95% CI: 0.24‐0.57). Table S1 shows the prevalence of new positive D7 reactions in each age group for each allergen group.

**Table 4:** Logistic regression analysis with new positive patch test reactions on day 7 as outcome

	Patients (N = 189)			
Factors^a^		n (%)	OR	95% CI
Sex	Male	56 (29.6)	1.00 (ref.)	
	Female	133 (70.4)	1.13	0.81‐1.58
Age (y)	<18	6 (3.2)	0.96	0.38‐2.42
	18‐30*	35 (18.5)	0.58	0.36‐0.93
	31‐45	48 (25.4)	0.73	0.47‐1.15
	46‐60	59 (31.2)	0.73	0.48‐1.12
	≥61	41 (21.7)	(ref.)	
Atopic dermatitis in lifetime	No	111 (58.7)	1.00 (ref.)	
	Yes	78 (41.3)	1.05	0.78‐1.43
Occupational dermatitis	No	153 (81.0)	1.0 (ref.)	
	Yes	36 (19.0)	1.35	0.92‐1.97
Allergen groups^b^	Allergens (n = 522)			
Metals (other = ref.)	Metals	147 (28.2)	0.94	0.76‐1.16
Preservatives (other = ref.)	Preservatives	113 (21.6)	0.82	0.66‐1.03
Fragrances (other = ref.)	Fragrances	95 (18.2)	0.99	0.78‐1.26
Rubbers (other = ref.)	Rubbers**	23 (4.4)	0.37	0.24‐0.57
Dyes (other = ref.)	Dyes	53 (10.2)	1.28	0.93‐1.76
Topicals (other = ref.)	Topicals**	72 (13.8)	2.60	1.92‐3.51
Corticosteroids (other = ref.)	Corticosteroids***	19 (3.6)	1.87	1.09‐3.21

CI, confidence interval; OR, odds ratio.

a Univariable.

b Univariable regression analysis controlled for age and sex.

* *P* = 0.025;

** *P* < 0.001;

*** *P* = 0.024.

### 3.3 Reaction strength

The reaction strength of the new positive D7 reactions and patch test results of the D3 reading are shown in Table 3. Of the 595 new positive D7 reactions in 189 patients, 548 (92.1%) were weak positive (+) and 46 (7.7%) were strong positive (++), especially to nickel sulfate, parthenolide, methylchloroisothiazolinone/methylisothiazolinone, colophonium, and epoxy resin. Only one extreme positive (+++) reaction was seen, namely, to epoxy resin. Of all new positive D7 reactions, 164 of 595 (27.6%) were regarded as doubtful and 4 of 595 (0.7%) were regarded as irritant at the D3 reading. Besides irritant and/or doubtful reactions, follicular reactions were seen to nickel sulfate (18 of 104 positive D7 reactions), cobalt chloride (2 of 25 positive D7 reactions, and ethylenediamine dihydrochloride (1 of 3 positive D7 reactions).

## 4 DISCUSSION

The present analysis provided estimates of the prevalence of new positive D7 patch test reactions in patients consecutively patch tested with the extended European baseline series and evaluated possible associated factors. Contact allergies would have been missed in 13.5% of the patch tested patients without a D7 reading.

In the current study, the prevalence of new positive D7 reactions was significantly associated with the allergen groups topicals and corticosteroids. In multiple studies addressing the added value of a late patch test reading, neomycin sulfate has been the most frequently reported allergen associated with new positive reactions at a late reading.^2^, ^7^, ^10^, ^16^ This is in agreement with our findings, in which neomycin sulfate was also the allergen with the highest proportion (81.5%) of new positive D7 reactions. A review by Macdonald et al on the characteristics of neomycin sulfate reported slow local absorption through the skin and slow local immunological reactivity. Furthermore, the possibility of a reservoir of neomycin being retained in the stratum corneum for a long time could be a factor contributing to the high share of new positive reactions at a late patch test reading.^17^ Although neomycin sulfate showed the highest proportion (new positive D7 reactions/total positive reactions), the total prevalence of new positive D7 reactions in all patch tested patients was only 0.7% (22/3209).

There have been conflicting results of studies on corticosteroids and the association with new positive D7 reactions. In our patients, corticosteroids were significantly associated with new positive D7 reactions, and gave a proportion of 28.4% new positive D7 reactions for all positively tested patients. A recent study by Chaudry et al reported that readings after D5 provided limited information, as no new reactions were seen to 0.01% budesonide, 1% clobetasol‐17‐propionate, 1% hydrocortisone‐17‐butyrate alcohol and 1% triamcinolone acetonide in 298 evaluated patients.^10^ It is interesting to note that budesonide was tested at a higher concentration of 0.1% in our baseline series. Davis et al evaluated 135 patients, and only two patients showed positive patch test reactions to corticosteroids, on D7 and D9. In their experience, the extended reading was of limited value.^18^ A study by Higgens et al presented 203 patients; no new positive reactions were observed to corticosteroids.^6^ Conversely, Madsen et al found a second reading on D6/D7 to be important for hydrocortisone‐17‐butyrate and budesonide.^7^ Because of the anti‐inflammatory effect, false‐negative patch test reactions at early readings can be expected when corticosteroids are tested at high concentrations.^19^, ^20^ Furthermore, corticosteroids induce local vasoconstriction with initial blanching and/or later vasodilatation. A possible positive patch test reaction could be confused with weak erythema resulting from vasodilatation. A further confounding issue is the “edge effect” phenomenon. In a classic positive patch test reaction, induration, erythema and infiltration cover the whole area. The edge effect is the manifestation of the skin reaction at the outer edge of the patch test site. It is believed that the anti‐inflammatory effect of high concentrations predominates in the centre, and low concentrations prevail at the periphery. An earlier study investigating the edge effect in patch testing with budesonide found that these reactions become more clearly positive at later readings, further supporting the need for a late reading.^21^ The differences in patch test concentrations between our study and recent studies, in addition to the challenges of patch test reading resulting from the anti‐inflammatory nature of corticosteroids, might explain the conflicting results in the literature.

In our cohort, a total of 607 patients reacted positively to nickel sulfate, and 104 (17.1%) of these reactions were new positive D7 reactions. It is interesting to note that 18 of these 104 new positive D7 reactions were labelled as follicular reactions at the corresponding D3 reading. Furthermore, nickel sulfate was the allergen with the highest number (n = 22) of doubtful or irritant reactions on D3 that became strong positive (++) by D7. A study by Thomas et al showed nickel sulfate to be the allergen with the second highest number of new positive D6 reactions. Of the 32 positive reactions, 11 (34.4%) appeared on D6.^11^ Ahlgren et al found a significantly higher frequency of new positive D7 reactions to metals, especially to mercury, nickel, gold, and cobalt.^5^ Chaudry et al reported late readings to be useful for metals, especially for gold sodium thiosulfate, which showed the highest prevalence of new positive reactions after D5.^10^ In our regression analysis, the group metals was not significantly associated with new positive D7 reactions as compared with other groups of allergens. However, our metal group consisted only of nickel sulfate, potassium dichromate and cobalt chloride from the European baseline series.

In the current analysis, the rubbers group was the only allergen group that was significantly less associated with new positive D7 reactions. Literature reports on the prevalence of rubbers showing new positive reactions at additional (late) patch test readings are scarce. A study by Madsen et al evaluated a second patch test reading on D6/D7 in patients who were tested with at least TRUE Test panels 1 and 2. Mercaptobenzothiazole and carba mix (together with paraben mix) were the only allergens that did not show new positive reactions at an additional reading, which is in agreement with our findings.^7^

### 4.1 Associated factors

In general, contact allergies may be associated with several patient characteristics. These include sex, age, and occupational dermatitis.^22^, ^23^, ^24^, ^25^, ^26^, ^27^, ^28^, ^29^, ^30^ Women tend to have a higher prevalence of contact allergies than men.^26^ Furthermore, several studies have evaluated the effect of age on the elicitation of contact allergy. Kwangsuksith et al reported a decrease in reactivity upon primary exposure to new antigens in older individuals, owing to senescence of the immune system.^26^ In our study, sex was not significantly associated with a higher prevalence of new positive reactions on D7. A study by Madsen et al reported significantly more new positive reactions in women and in patients aged >40 years.^7^ In the current study, an age of 18 to 30 years were significantly less associated with new positive D7 reactions than an age of ≥61 years. However, allergens could be a confounding factor. The allergen with the highest proportion of new D7 reactions was neomycin sulfate. The use of neomycin sulfate in topicals in The Netherlands was reduced years ago. Consequently, it was mainly the elderly who were exposed, which could explain the lower proportion of new positive reactions in the age group 18 to 30 years. Logistic regression analysis was controlled for age and sex. Nevertheless, we found a significant association for age in all subgroups of allergens analysed. Thus, the effect of age can be only partly moderated by the exposure to allergens. For patients suffering from occupational dermatitis, it could be hypothesized that sensitization occurs earlier because of a high probability of impaired barrier function of the skin resulting from concomitant exposure to irritants, allowing for more rapid penetration of the contact allergen.^23^, ^30^ In the current analysis, no differences were observed for the presence of occupational dermatitis between patients with new positive D7 reactions and patients with positive D3 reactions. Concerning the overall prevalence of contact allergy in atopic vs non‐atopic dermatitis patients, Johansen et al concluded that there were no significant differences.^31^ In the current analysis, the prevalence of new positive D7 reactions was also comparable between atopic and non‐atopic dermatitis patients.

### 4.2 Strength and limitations

The added value of a D7 reading has been underscored by previous studies, but these investigations have mostly been performed on small populations, whereas the current study was performed on a large sample. A novel aspect was the investigation of patient characteristics in relation to late positive reactions. One limitation is the retrospective nature of the study. Furthermore, it is challenging to compare publications about new (delayed) positive patch test reactions at additional (late) patch test readings. Patch test materials and concentrations, vehicles, techniques and patch test interpretation differ between studies. Furthermore, variations in terminology and day of the additional patch test reading (D5‐D9) do not always match in the compared studies.

## 5 CONCLUSION

The results of the present analysis support the importance of an additional late patch test reading on D7. Especially for topicals and corticosteroids, the share of new D7 reactions is high. Our results show that, in patients tested with True Test panel 1 and 2 and additional allergens, 13.5% of positive reactions are missed if no D7 reading is performed. Therefore, it is recommended to perform a D7 patch test reading to detect new, clinically relevant positive patch test reactions.

## CONFLICTS OF INTEREST

There was no funding and the authors have no conflicts of interest to report.

## Supporting information


**TABLE S1** Relative number of new positive D7 reactions in each age group for each allergen group.
