# Supplementary material for: New positive patch test reactions on day 7—The additional value of the day 7 patch test reading
Source: Contact Dermatitis. 2019 Jun 11;81(4):280–7. doi: 10.1111/cod.13322 (PMC6771944; doi:10.1111/cod.13322)
Supplement: Supplementary file 1 — TABLE S1 Relative number of new positive D7 reactions in each age group for each allergen group. [file COD-81-280-s001.docx]

**Supplemental information:**

|  |  | < 18 years | 18 – 30 years | 31 – 45 years | 46 – 60 years | ≥ 61 years |
| --- | --- | --- | --- | --- | --- | --- |
| New positive D7 reactions to | N | N (%) | N (%) | N (%) | N (%) | N (%) |
| Metals | 147 | 3 (2.0) | 24 (16.3) | 43 (29.3) | 50 (34.0) | 27 (18.4) |
| Preservatives | 113 | 0 (0.0) | 14 (12.4) | 30 (26.5) | 40 (35.4) | 29 (25.7) |
| Fragrances | 95 | 1 (1.1) | 30 (31.6) | 19 (20.0) | 27 (28.4) | 18 (18.9) |
| Rubbers | 23 | 1 (4.3) | 7 (30.4) | 4 (17.4) | 6 (26.1) | 5 (21.7) |
| Dyes | 53 | 2 (3.8) | 12 (22.6) | 15 (28.3) | 13 (24.5) | 11 (20.8) |
| Topicals | 72 | 1 (1.4) | 8 (11.1) | 12 (16.7) | 29 (40.3) | 22 (30.6) |
| Corticosteroids | 19 | 0 (0.0) | 2 (10.5) | 6 (31.6) | 6 (31.6) | 5 (26.3) |

**Supplemental table.** Relative number of new positive D7 reactions in each age group for each allergen group
